# Multilayer thin films with compositional PbZr_0.52_Ti_0.48_O_3_/Bi_1.5_Zn_1.0_Nb_1.5_O_7_ layers for tunable applications

**DOI:** 10.1038/srep10173

**Published:** 2015-05-11

**Authors:** Shihui Yu, Lingxia Li, Weifeng Zhang, Zheng Sun, Helei Dong

**Affiliations:** 1 School of Electronic and Information Engineering, Tianjin University, Tianjin 300072, P. R. China; 2 Key Laboratory of Photovoltaic Materials of Henan Province and School of Physics and Electronics, Henan University, Kaifeng 475004, P. R. China

## Abstract

The dielectric properties and tunability of multilayer thin films with compositional PbZr_0.52_Ti_0.48_O_3_/Bi_1.5_Zn_1.0_Nb_1.5_O_7_ (PZT/BZN) layers (PPBLs) fabricated by pulsed laser deposition on Pt/TiO_2_/SiO_2_/Si substrate have been investigated. Dielectric measurements indicate that the PZT/BZN bilayer thin films exhibit medium dielectric constant of about 490, low loss tangent of 0.017, and superior tunable dielectric properties (tunability = 49.7% at 500 kV/cm) at a PZT/BZN thickness ratio of 3, while the largest figure of merit is obtained as 51.8. The thickness effect is discussed with a series connection model of bilayer capacitors, and the calculated dielectric constant and loss tangent are obtained. Furthermore, five kinds of thin–film samples comprising single bilayers, two, three, four and five PPBLs were also elaborated with the final same thickness. The four PPBLs show the largest dielectric constant of ~538 and tunability of 53.3% at a maximum applied bias field of 500 kV/cm and the lowest loss tangent of ~0.015, while the largest figure of merit is 65.6. The results indicate that four PPBLs are excellent candidates for applications of tunable devices.

Ferroelectric materials are attractive materials for the applications in tunable devices at radio and microwave frequencies due to their high dielectric constant and agile feature under an external electric field^1^,^2^,^3^. A potential application of ferroelectric materials is in microwave tunable devices, including tunable mixers, delay lines, filters, and phase shifters for steerable antennas^4^,^5^,^6^,^7^. Among the various tunable ferroelectric materials, lead zirconate titanate (PbZr_1–x_Ti_x_O_3_, PZT) thin films have emerged as leading candidates for such applications due to their highly nonlinear dielectric response to an applied electric field^8^,^9^. However, due to the oxidation state of titanium being easily reduced from Ti^4+^to Ti^3+^^10^,^11^,^12^, the dielectric tunable properties of these titanium–containing ferroelectric thin films usually undergo degradation (dielectric loss increasing and figure of merit decreasing). The high dielectric loss and low figure of merit (FOM) is a crucial limitation for practical utilizations^13^,^14^. In order to reduce the dielectric loss of pure PZT thin films, great efforts have been made in the past decades, such as doping with various elements (La^2+^, Mn^2+^, Sr^2+^etc.)^15^,^16^,^17^,^18^,^19^. For the thin films, in addition to the above–mentioned methods, many strategies, such as post thermal annealing, uses of buffer layers and/or oriented single–crystal substrates, and compositional gradation^20^,^21^,^22^,^23^,^24^, have been adopted to reduce the dielectric loss and modify the dielectric constants. Among various approaches, ferroelectric/dielectric layered composite is proved to be a flexible and efficient way^25^,^26^,^27^. Nonetheless, the dielectric layer with low dielectric constant inclines to consume a majority of bias voltage, resulting in a deteriorated tunable performance of the composite.

Recently, cubic pyrochlore bismuth zinc niobate (Bi_1.5_Zn_1.0_Nb_1.5_O_7_, BZN) dielectric thin films have gained a great deal of attention in tunable applications because of their very low dielectric loss tangents and certain tunability^28^,^29^. Compared to other low–loss dielectrics with low dielectric constant, TiO_2_ (<50)^30^ and MgO (~10)^31^, BZN offers much higher dielectric constant (~200) with additional tunability^29^, thus sharpening the overall dielectric performance as a whole. We suggested that the heterolayered structure of PZT thin films with a BZN layer would decrease the overall dielectric loss and improve the FOM, which is very important for practical application. However, no studies have been reported on PZT/BZN heterolayered thin film metal-insulation-metal (MIM) configuration. In this paper, we report the preparation and characterization of PbZr_0.52_Ti_0.48_O_3_/ Bi_1.5_Zn_1.0_Nb_1.5_O_7_ (PZT/BZN) bilayer thin films and the multilayer thin films with compositionally PZT/BZN layers (PPBLs) deposited by pulsed laser deposition, using two individual PZT and BZN targets. Our results show that the four PPBLs with a PZT/BZN thickness ratio of 3 demonstrate a lower loss tangent while retaining relatively high tunability, making them a promising candidate for integrated device applications.

## Results

### PbZr_0.52_Ti_0.48_O_3_/Bi_1.5_Zn_1.0_Nb_1.5_O_7_ bilayer thin films

The thickness ratio x is defined as


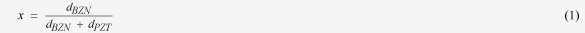


where *d*_*BZN*_ is the thickness of BZN layer and *d*_*PZT*_ is the thickness of PZT layer.

**Figure 1:**
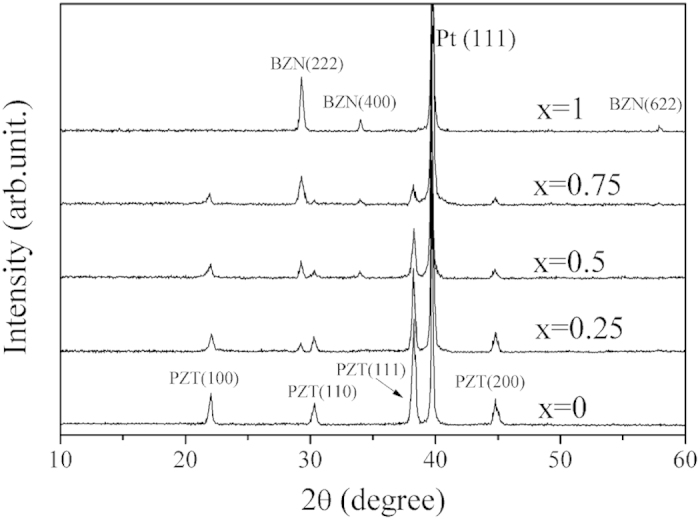
The XRD patterns of the PZT thin films, BZN thin films and the PZT/BZN bilayer thin films.

The X-ray Diffraction (XRD) patterns of the PZT thin film, BZN thin film and the PZT/BZN bilayer thin films are shown in Fig. 1. The X–ray diffraction patterns of PZT thin films show single–phase perovskite peaks^32^, and that of BZN thin films show cubic pyrochlore peaks^29^, no extra peaks were observed in either thin films. The PZT/BZN bilayer thin films were composed of a perovskite PZT phase and a cubic pyrochlore BZN phase. No impurity phases can be detected with PZT addition on the BZN layer, which indicates that no measurable reaction occurred between PZT and BZN layers. With an increase in thickness ratio *x*, the intensities of the BZN thin film phase peaks become more intense and sharper. This could be explained, as the crystallinity of the thin films is improved and the grain size becomes larger with the BZN thin film thickness increases. Similarly, the intensities of the PZT thin film phase peaks decrease as the thickness ratio *x* increases. Meanwhile, the PZT thin films have a preferred orientation along the (1 1 1) direction due to the influence of the (1 1 1) phase of platinum.

**Figure 2:**
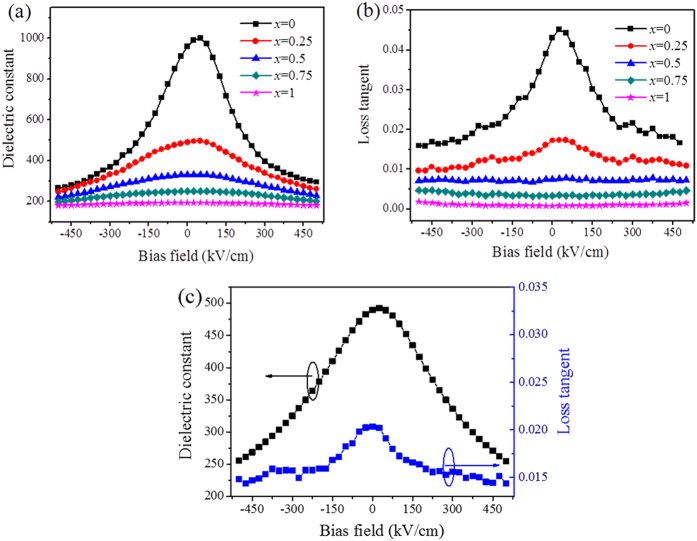
(**a**) The dielectric constant (ε) and (**b**) loss tangent (*tan δ*) of PZT/BZN bilayer thin films with different thickness ratios as a function of bias field at 100 kHz. (**c**) The dielectric constant and loss tangent (*tan δ*) PZT/BZN bilayer thin films with Pt/PZT/BZN/Pt capacitors as a function of bias field at 100 kHz.

The electric field dependence of dielectric constant and loss tangent of the PZT/BZN bilayer thin films with different thickness ratios (*x*) were characterized at room temperature. Fig. 2a shows the dielectric constant vs. applied bias field for the thin films with different thickness ratios (*x*). The curves of dielectric constant and the loss tangent varying with applied bias field were calculated from the capacitance–voltage (C–V) characteristics which were measured at the frequency of 100 kHz with a small ac signal amplitude of 0.1 V, while the applied bias voltage was swept from negative bias voltage (–40 V) to positive bias voltage (+40 V). As shown in Fig. 2a, the dielectric constant of the pure PZT thin films is ~960 at zero bias field. As predicted, the dielectric constant of the PZT/BZN bilayer thin films decreases with the increase of thickness ratio (*x*) at zero bias field. The dielectric constant of all the thin films nonlinearly decreases with increasing applied bias field. The tunability (*T*) is calculated by the following formula:


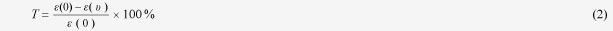


where *ε*(0) is the measured dielectric constant at zero bias field and *ε*(υ) is the dielectric constant at a certain bias field^33^. In the meantime, the PZT/BZN bilayer thin films demonstrate gradually deteriorate tunability performance at applied bias field of 500 kV/cm, which is about 72.1%, 49.7%, 33.5%, 20.8% and 6.5% for the thickness ratios (*x*) of 0, 0.25, 0.5, 0.75 and 1, respectively. The tunability and dielectric constant decrease with increasing thickness ratio (*x*) should be ascribed to the low tunability and low dielectric constant of the BZN thin films. The dielectric loss tangent vs. applied bias field for the thin films with different thickness ratios (*x*) is shown in Fig. 2b, the loss tangent (*tan δ*) of the PZT/BZN bilayer thin films as well as decreases with the rise of thickness ratio (*x*). Moreover, the *tan δ* of PZT/BZN bilayer thin films change little with the change of applied bias fields. The reduction of loss tangent can be ascribed to the presence of BZN phase in PZT thin films. The BZN thin films have lower loss tangent than PZT thin films, so the *tan δ* decrease with the BZN layer thickness increasing.

An asymmetric characteristic of the applied bias field dependent dielectric constant (as well as the loss tangent) of the thin films was observed, which may be attributed to the different interface conditions between top and bottom electrodes. As discussed by many researchers, an interfacial layer exists between the thin film and the electrode, and a built–in electric field could be formed at the interfacial layer^34^,^35^,^36^. For the Au/ dielectric/Pt capacitors, there are two different interfacial layers formed on the top and bottom electrodes, and then the built–in electric fields could not counteract each other. So the built–in electric field should be the origin of the asymmetric behaviour, because the applied bias electric field on the thin film capacitor must overcome the effect of the built–in electric field. In addition, the oxygen vacancies at the PZT/Au interface leading to the trapping of electrons when positive voltage is applied can also be a possible reason of asymmetric characteristic^37^. In order to verify the origin of the built–in electric field, the Pt/PZT(600 nm)/BZN (200 nm)/Pt capacitors were prepared. Fig. 2c displays the dielectric constant and loss tangent of the samples as a function of applied bias field. The asymmetric behavior of bias field dependent permittivity was much improved, which indicates that the build-in electric field results from the unsymmetrical structure of the top and bottom electrodes.

**Figure 3:**
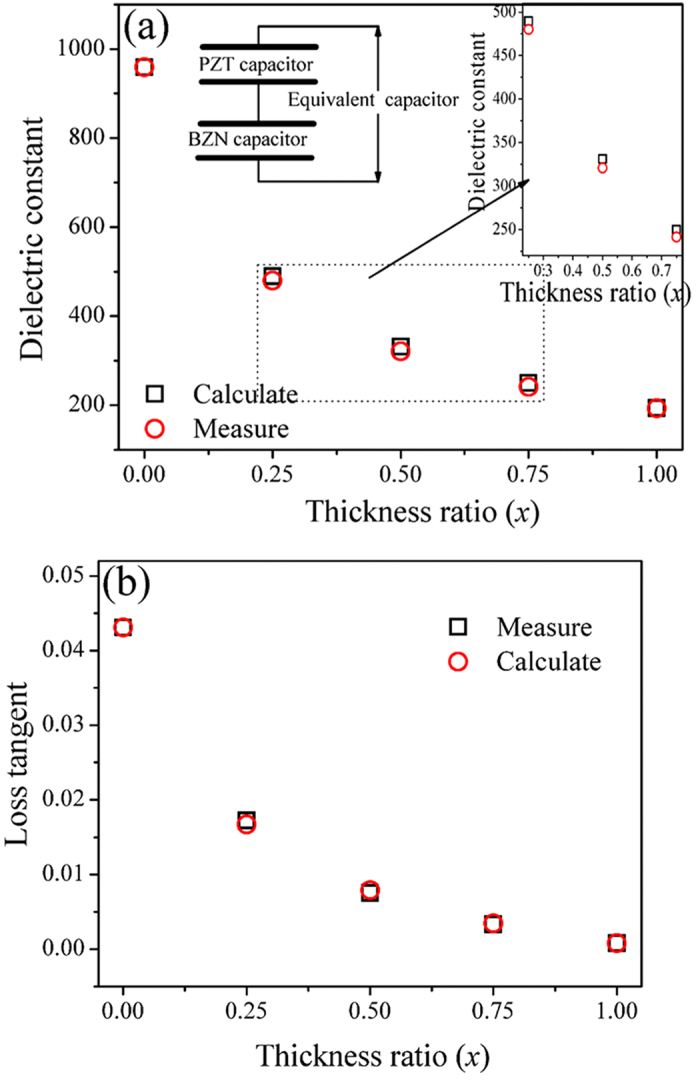
Simulations of thickness ratio dependent (**a**) dielectric constant and (**b**) loss tangent of PZT/BZN thin films using the series connection model of bilayer capacitors; Inset in figure 3a is the series connection model of ideal capacitors.

The measured capacitance of the MIM structure is the series connection of the capacitance of the PZT and BZN layer if the bias field is applied along the thickness direction (the series connection model of ideal capacitors is shown in Fig. 3a). Thus the measured total capacitance (*C*) can be evaluated from the series connection of the PZT capacitance and the BZN capacitance as follows^38^


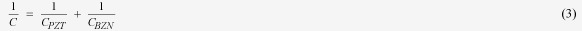


So,


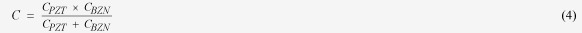


where *C*_*PZT*_ is the capacitance of PZT thin films and *C*_*BZN*_ is the capacitance of BZN thin films. Given the same area, the average dielectric constant, *ε*_*av*_, of the PZT/BZN bilayer thin films can be expressed as:


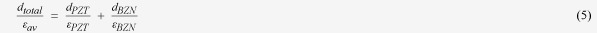


Combined with Eqs (1) and (4),


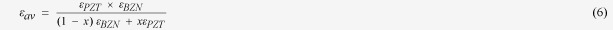


where *d*_*total*_, *d*_*PZT*_, and *d*_*BZN*_ represent the thickness of the total thin film, PZT and BZN thin film, respectivley. *ε*_*PZT*_, *ε*_*BZN*_ is the dielectric constant of the single–layer PZT and BZN in our experiment, which is ~960 and ~193, respectively. The average dielectric constant calculated from Eq. (6) as well as measurements of the PZT/BZN bilayer thin films under zero bias field are presented in Fig. 3a. The average dielectric constant calculated from Eq. (6) exhibit a similar trend to the results of the experiment. As BZN is a low dielectric layer, the decreased dielectric constant of the PZT/BZN bilayer thin films with increasing BZN thickness can be partially understood by Eq. (4). However, it is also seen that the dielectric constant of the simulated is a little lower than that of the measurements, the difference between the simulation and measurement can be explained through Maxwell–Wagner model. In addition to the contribution as predicted by Maxwell–Wagner model^39^, the dielectric constant of PZT/BZN heterostructures is observed to show contribution due to the strain induced polarization, due to mismatch of lattice parameters of individual layers. This is commonly understood as super lattice effect^40^,^41^. Therefore instead of Eq. (6), the average dielectric constant of PZT/BZN bilayer thin films follows following inequality


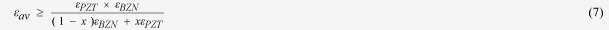


Considering the series connection model of ideal capacitors, the power consumption of the PZT/BZN bilayer thin films should be the sum of the consumption of the PZT and BZN thin films, as follows:


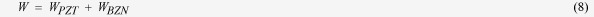


where *W* is the total power consumption of PZT/BZN bilayer thin films, *W*_*PZT*_ and *W*_*BZN*_ are the power consumption of the PZT and BZN thin films, respectively. With dielectric loss tangent, Eq. (8) can be written as


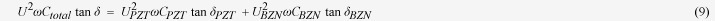


where *U* is the total voltage applied in PZT/BZN bilayer thin films, *U*_*PZT*_ and *U*_*BZN*_ are the voltages applied in the PZT and BZN thin films, respectively. *ω* is the angular frequency, *tan δ*, *tan δ*_*PZT*_ and *tan δ*_*BZN*_ are the loss tangents of the PZT/BZN bilayer thin films, PZT and BZN thin films, respectively.

According to Gauss’s Law


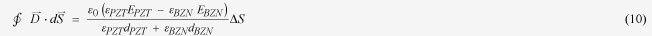


So


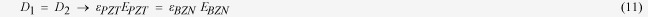


Where *E*_*PZT*_ and *E*_*BZN*_ are the electric fields applied in the PZT and BZN thin films, respectively.

In addition


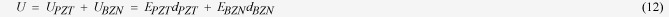


Combined with Eqs (3), (10) and (11), the dielectric loss tangent (*tan δ*) (accordingly, polarization loss) of the total PZT/BZN bilayer thin films can be expressed as


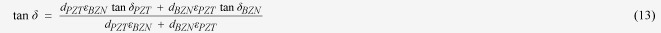


The simulation results and the measured loss tangent of the PZT/BZN bilayer thin films at zero bias field as a function of the thickness ratio are plotted in Fig. 3b. In the simulation, *tan δ*_*PZT*_ and *tan δ*_*BZN*_ is ~0.043 and ~0.001, respectively. Fig. 3b shows that the experimental results are in good accordance with the simulation results, indicating that the relationship between the loss tangent and the thickness ratio comply with the theory, wherein the loss tangent decreases as the thickness ratio increases.

Both the dielectric tunability and dielectric loss tangent of the PZT/BZN bilayer thin films decreased with increasing thickness ratio (*x*). Obtaining the thin–film material for the best device performance is based on the optimal tradeoff between the tunability and dielectric loss tangent. The figure of merit (FOM), defined as the ratio of the relative tunability to the loss tangent, is commonly used to reflect the trade–off between dielectric tunability and loss tangent^2^. The FOM is defined as.


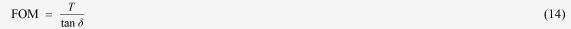


which is a balanced combination of tunability and dielectric loss. The figures of merit obtained are 45.6, 51.8, 47.2, 44.1, and 40.6 for the thickness ratio (*x*) of 0, 0.25, 0.5, 0.75 and 1, respectively. The largest FOM (51.8) is obtained when the thickness ratio (*x*) is 0.25, at which the tunability and dielectric loss tangent exhibit an optimum balance.

### Multilayer thin films with compositional PbZr_0.52_Ti_0.48_O_3_/Bi_1.5_Zn_1.0_Nb_1.5_O_7_ layers

**Figure 4:**
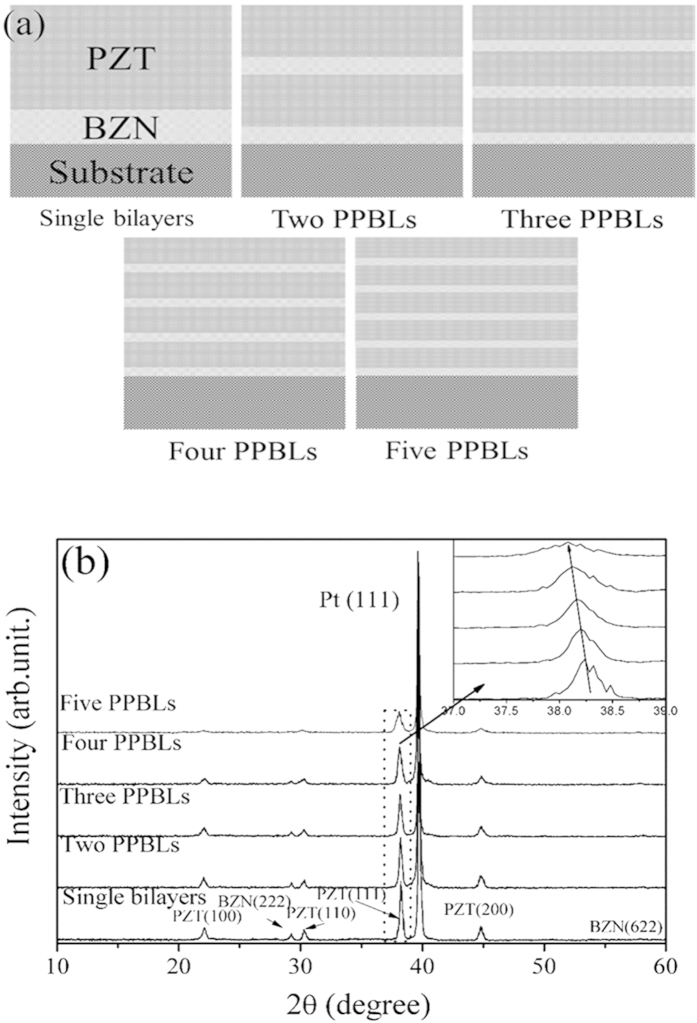
(**a**) The schematic configurations of the multilayer thin films with periodic compositional PZT/BZN layers. The samples were represented as single bilayers, two, three, four and five PPBLs, respectively. (**b**) X–ray diffraction patterns for the multilayer thin films with PPBLs.

According to the above results, we obtained that the PZT/BZN bilayer thin films exhibited the largest figure of merit FOM at the thickness ratio (*x*) of 0.25. So we decided to fix the thickness ratio (*x*) at 0.25 to investigate the properties of the multilayer thin films with compositional PZT/BZN layers (PPBLs). The total thickness of all the multilayer thin films was controlled around 800 nm by the series of compositionally PZT/BZN layers. The schematic configurations of the final thin films are shown in Fig. 4a, and the samples are represented as single bilayers, two PPBLs, three PPBLs, four PPBLs and five PPBLs, respectively. In each individual layers, the thicknesses of the compositionally PZT/BZN layers are about 800, 400, 270, 200 and 160 nm for the five samples, respectively.

Figure 4b shows the XRD patterns of the multilayer thin films with PPBLs. All the multilayer thin films were composed of a perovskite PZT phase and a cubic pyrochlore BZN phase. No impurity phases can be detected with BZN addition onto the PZT layer, which excludes interdiffusion between the PZT/BZN layers. Furthermore, all the diffraction peaks shift together with increasing number of the PPBLs, indicating the reduction of the difference in the lattice parameters among different layers by the modulation of internal strain. As the number of layer increased, the intensities of all the diffraction peaks decreased. The more the number of PPBLs, the thinner the individual layer thickness, the small thickness is detrimental for the growth of grain.

**Figure 5:**
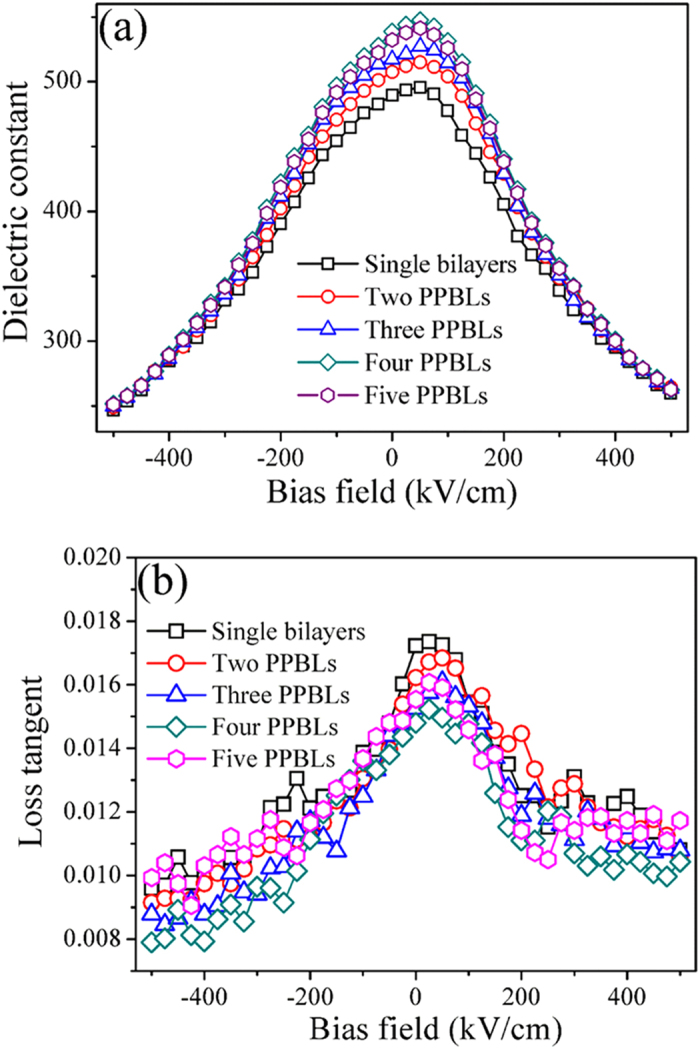
Electric field dependence of the (**a**) dielectric constant and (**b**) loss tangent of the multilayer thin films with PPBLs at 100 kHz.

The applied bias field dependent dielectric constant of the multilayer thin films with PPBLs was measured. It turns out that a large tunability of 50%–55% can be achieved in a repeatable manner. Figure 5 shows the dielectric constant (ε) and loss tangent (*tan δ*) of the multilayer thin films as a function of applied bias field at 100 kHz. The dielectric constant at zero bias field are ~490, ~507, ~517, ~538 and ~532 for the single bilayers, two, three, four and five PPBLs, respectively, and the values of dielectric loss tangents for all multilayer thin films are below 0.015. When the number of PPBLs is less than 4, the dielectric constant of the multilayer thin films gradually increases with the increasing the number of PPBLs. In other words, multilayer thin films with thinner individual layer within the periodic structure have a higher resultant dielectric constant value, as also previously observed by Shen group^42^ and Zhao group^43^. It’s reported that the interfaces between each layers have heterogeneous compositions, and a space charge layer has been introduced in this micro–region^42^. According to Maxwell–Wagner model^39^, the dielectric constant of the thin films with multilayer structure increases. However, the dielectric constant began to decrease, when the number of PPBLs is 5. Which can be ascribed to the poor crystallinity (as shown in Fig. 4). The tunability calculated from Eq. (2) are 49.7%, 51.0%, 51.8%, 53.3%, 52.8% for the single bilayers, two PPBLs, three PPBLs, four PPBLs and five PPBLs, respectively. The tunability for the multilayer thin films follows similar trend to that dielectric constant as a function of number of PPBLs. These results can be qualitatively interpreted by the phenomenological theory of Devonshire and the simplified expression proposed by Johnson^44^


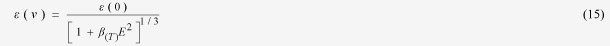


where *β*_*(T)*_ is a temperature–dependent constant given by


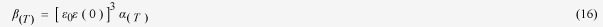


where *α*_*(T)*_ is a temperature–dependent constant that provides information on the degree of anharmonic contributions of the polarization to the free energy^45^. As shown in Fig. 5a, the *ε–E* curves can be fitted well over the whole electric field range. Combined with Eqs (1), (15) and (16), the dielectric tunability can be written as


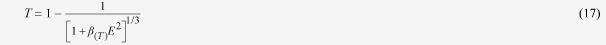


Eq. (17) demonstrates that the larger the *β*_*(T)*_ value, the larger the dielectric tunability would be. Therefore, it is predicted that the higher the value of dielectric constant at zero electric field, the larger the tunability would be.

From a device application point of view, thin films for microwave tunable device applications should have a certain value of dielectric constant (~500), a large tunability (as large as possible) and a low loss tangent (0.02 or less)^14^,^46^. High dielectric losses, corresponding to an attenuation of the microwave signal, result in inferior device performance. Specifically, for phase shifter applications, a low dielectric loss tangent is desirable to reduce the insertion loss and, hence, increase the phase shift per decibel of loss^47^. This multilayer thin films with four periodic compositional PZT/BZN layers are very reasonable for practical device applications.

**Figure 6:**
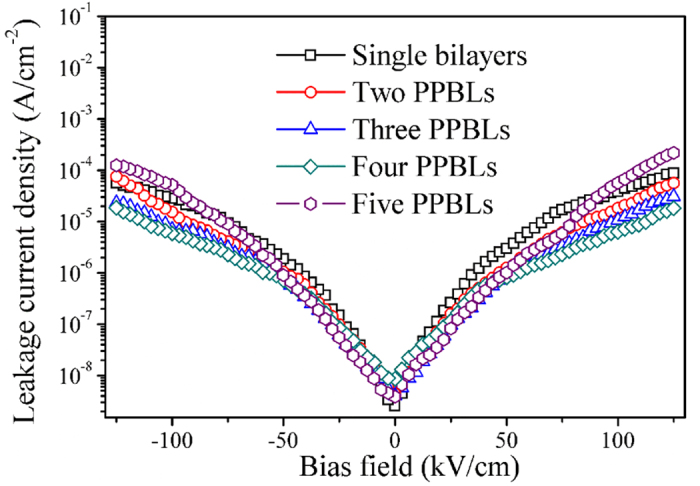
Leakage current density of the multilayer thin films with PPBLs.

Furthermore, the dielectric loss tangent of multilayer thin films decreases as the number of PPBLs increase from 1 to 4. However, with a further increase of the number of PPBLs to 5, the dielectric loss increase. The dielectric loss tangent for the four PPBLs was found to be reduced by ~15% with respect to that of the single bilayers. The factors influencing the dielectric loss are quite complicated. However, at low frequency considered in this study, the overall dielectric loss is caused by the polarization loss (it is strongly affected by space dipole determined by interface conditions and defects in the ferroelectric thin films) and leakage conductance loss^48^,^49^. The leakage conductance loss was evaluated by leakage current measurements. And, leakage current is also one of the limiting factors for the suitability of a dielectric material for tunable device applications^50^. Figure 6 shows the leakage current density–electric field characteristics of the multilayer thin films. The leakage current density of the multilayer thin films increased gradually with an increase in the applied bias field. For an applied bias field of 125 kV/cm, the leakage current density decreased monotonically with increasing number of the PPBLs. At an applied bias field of 125 kV/cm, the value of leakage current density is 8.6 × 10^−5^ A/cm^2^ for single bilayers. As the number of PPBLs increases to four, the value of leakage current density decrease to 1.8 × 10^−5^ A/cm^2^ at the same applied bias field, which is almost one fifth of the value for the single bilayers. Thus, the results show that, due to the reduction of leakage current density, the design of periodic structure did contribute to the decrease in the dielectric loss tangent.

The figures of merit FOM, which were determined according to Eq. (14), are 51.8, 55.2, 57.9, 65.6, 53.1 for the multilayer thin films with single bilayers, two PPBLs, three PPBLs, four PPBLs and five PPBLs, respectively. The largest value of figure of merit is obtained when the number of the PPBLs is four. The results suggest the enhancement of dielectric constant and tunability could be obtained by the design of periodic structure.

In order to further study the relationship of between the dielectric tunability and applied bias field, a linear expression between tunability and applied bias field square (*E*^2^) can be derived as follows:


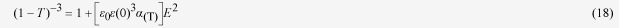


**Figure 7:**
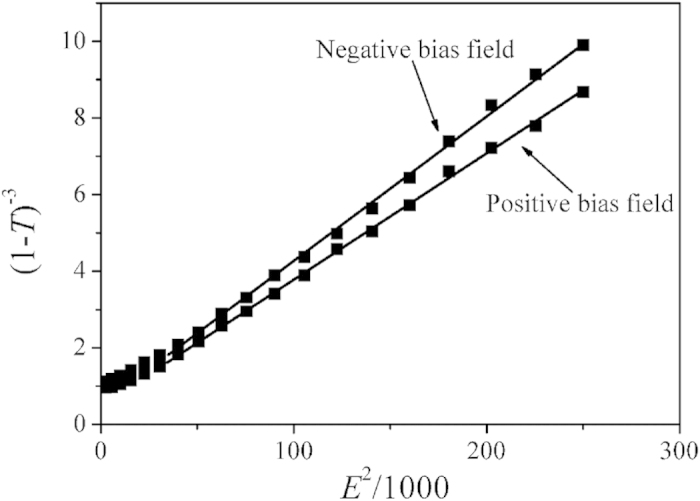
The (1−*T*)^–3^ versus *E*^2^/1000 for the multilayer thin films with four PPBLs.

Figure 7 shows the *E*^2^ dependence of (1*−T*)^–3^ It can be found that the experimental data are in good accord with Eq. (18) when *E* > 150 kV/cm, indicating that the dielectric tunable performance is mainly ascribed to the intrinsic lattice phonon polarization^51^,^52^. When *E* < 150 kV/cm, there is largely deviation between experimental data and Eq. (5), indicating that the reorientation^53^ of the nanopolar clusters^54^ (which is the typical characteristic of ferroelectric relaxor) and (or) the nanononpolar clusters (in antiferroelectric phase) plays an important role in the tunability property of multilayer thin films, in addition to the intrinsic lattice phonon polarization^52^.

The most straightforward description of the dielectric response of ferroelectrics is given by the conventional Landau theory and is based upon an expansion of the Helmholtz free energy F with respect to the vector macroscopic polarization P. For the situation where the polarization is collinear with the macroscopic electric field E in the material^25^,^55^, the first two terms of this expansion can be expressed as^25^


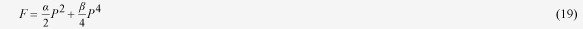


here, *F* is the Helmholtz free energy and *P* is the vector macroscopic polarization. Using ∂F/∂P = E, then leads to a relation between the polarization and electric field:


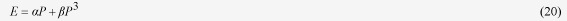


Eq. (20) enables us to present the relative dielectric constant of the material in the form:


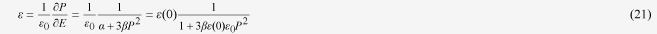


where *ε*_*0*_ is the vacuum dielectric constant and *ε*(0) is the dielectric constant at zero bias field, and *α = 1/ε*_*0*_*ε*(0). This expression describes the dielectric permittivity both in the absence of a bias field and under it. Accordingly, the absolute dielectric tunability is given by


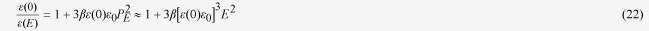


where *P*_*E*_ is the polarization induced by the bias field. Accordingly, there is a linear relationship between *ε(0)/ε(E)* and *E*^2^ with a gradient of 3*β*[*ε*(0)*ε*_0_]^**3**^. From the expression of gradient, the coefficient of the dielectric nonlinearity *β* can be extracted. Table 1 summarizes the tunable characteristics and gives the coefficient of the dielectric nonlinearity of the four PPBLs in this study. The values of *β* in the PZT thin films and BZN thin films were also calculated, respectively. The value 4.35 × 10^9^*J/C*^*4*^*m*^*5*^ of the four PPBLs is larger than that 2.11 × 10^9^ *J/C*^*4*^*m*^5^ of BZN thin films and 3.32 × 10^9^ *J/C*^*4*^*m*^*5*^ of PZT thin films.

**Figure 8:**
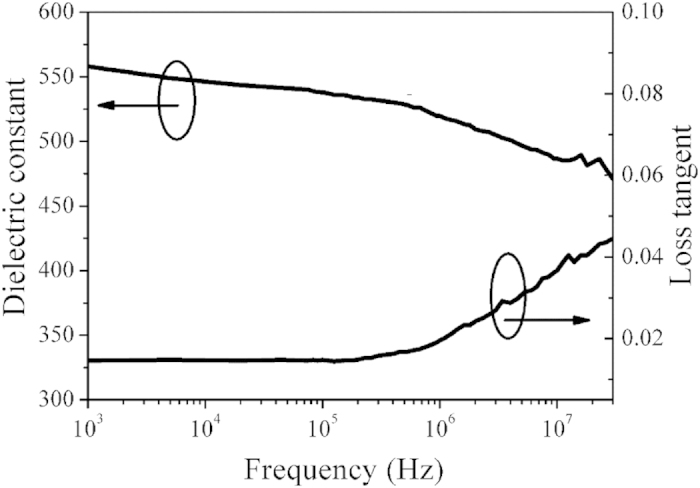
Dielectric constant and loss tangent of multilayer thin films with four PPBLs as a function of frequency ranging from 1 kHz to 10 MHz.

Dielectric constant (ε_r_) and loss tangent (*tan δ*) are important for the functioning of any dielectric thin films–based devices. The dielectric constant and dielectric loss were measured by applying a small ac signal of 0.1 V amplitude as a function of frequency in the range of 1 kHz–10 MHz at room temperature. The frequency dependence on the dielectric constant and loss tangent of the multilayer thin film with four PPBLs are shown in Fig. 8. A monotonic decrease in dielectric constant is found in the measured frequency range of 100 Hz–10 MHz. The phenomenon has been observed in many ferroelectric thin films^56^,^57^,^58^. There are many kinds of polarization (such as defect dipole polarization, ion polarization, electronic polarization, etc.) contributing to the dielectric constant of the thin films. In the lower frequency region the dielectric constant is high due to that all the defect dipole polarization can keep up with reversal of external electric field. With increasing frequency, the other polarization cannot gradually keep up with reversal of external electric field and the significance of these polarization loss gradually, which contributes to the decrease of dielectric constant^59^. It also was observed that loss tangent for the multilayer thin films remained essentially constant around 0.008 in the frequency range of 1 ~ 200 kHz. As the frequency increases beyond 200 kHz, the loss tangent increases with frequency. A strong increase of loss tangent is observed at a high frequency of >1 MHz, which is extrinsic in nature due to the resonance of the equivalent circuit. At frequencies on the order of a MHz, the stray inductance of the contact and the leads may induce L−C resonance 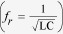
60, where L and C are the inductance and the capacitance of the equivalent circuit, respectively. For the thin films having capacitances on the order of 100 pF or so (as in the present case), a stray in-ductance on the order of a few μH can induce resonance in the MHz range. This seems to be the reason for the strong increase of *tan δ* at the high frequency of 1 MHz since such resonance behavior starts around 1 MHz. In the measurement range, the dielectric loss tangent is less than 0.05, however, for potential microwave applications, the dielectric constant and loss at microwave frequencies should be further explored.

## Discussion

The multilayer films with compositional PbZr_0.52_Ti_0.48_O_3_/Bi_1.5_Zn_1.0_Nb_1.5_O_7_ (PZT/BZN) layers (PPBLs) have been fabricated by pulsed laser deposition on Pt/TiO_2_/SiO_2_/Si substrate. We investigated the structural, dielectric properties and tunability of multilayer films with various thickness ratio (*x*) and number of PPBLs. As the thickness ratio (*x*) increases, both the dielectric constant and tunability decrease. As the number of PPBLs increases from 1 to 4, the dielectric constant and tunability increases. In brief, the thickness of BZN layer and number of PPBLs have important role in controlling the dielectric properties and tunability the multilayer films. The multilayer films with PPBLs are obtained with the highest figure of merit of 65.6 with a dielectric constant of ~538 and a tunability of 53.3% (500 kV/cm) and a loss tangent of ~0.015 at the thickness ratio (*x*) of 0.25 and PPBLs number of four. The medium dielectric constant, high tunability and low loss tangent of the dielectric constant suggest that multilayer thin films with four PPBLs have potential application for tunable microwave device applications.

## Methods

The thin films of PbZr_0.52_Ti_0.48_O_3_ (PZT) and Bi_1.5_Zn_1.0_Nb_1.5_O_7_ (BZN) were deposited on Pt/Ti/SiO_2_/Si substrates using ceramic PZT and BZN targets by pulse laser deposition using a KrF excimer laser with a wavelength of 248 nm, a pulse width of 30 ns and a repetition rate of 5 Hz. The PZT ceramic with 25 at.% lead excess and 6 at.% titanium excess was used as PZT target. The BZN ceramic with 10 at.% bismuth excess and 5 at.% zinc excess was used as BZN target. Prior to deposited, the vacuum chamber was evacuated to a base pressure of lower than 3.0 × 10^−4^ Pa. The substrates are fixed at an on–axis distance of 5 cm from the target and the deposition is done. The laser radiation is impinged on the target at 45° with respect to normal in a dynamic flow of oxygen. Before irradiations, the deposition chamber is evacuated down to a base pressure of 3 × 10^−4^ Pa. The substrate temperature was 700 °C and the pressure of the ambient oxygen gas was 10 Pa during deposition for all BZN and PZT thin films. The individual layers were grown from the different targets without breaking the vacuum in order to eliminate the formation of any surface layers, which might extrinsically affect the thin film properties. The total thickness of all the thin films was controlled around 800 nm. The thickness of individual layers was controlled by the time of deposition. After deposition, a post annealing was performed for 30 min in 1 atm oxygen at 500 °C.

### Characterization

The crystallinity was determined by X–ray diffraction using a (Rigaku D/MAX–RB, Akishima, Tokyo, Japan) system equipped with a Cu–Kα radiation source(1.542 Å). The thickness was measured by Alpha–Step D–100 profilometer (KLA–Tencor, California, USA). For electrical measurement, Au top electrodes with 0.2 mm in diameter were patterned by lift–off process to form the metal–insulator–metal type capacitors. The dielectric properties and tunability were measured at room temperature by LCR analyzer (TH2828S, Tonghui Electronics, Shenzhen, China) and precision LCR meter (Aglient 4285A, Santa Clara, California, USA). The leakage current density of the thin films was measured by Aglient 4339B High Resistance Meter (Santa Clara, CA, USA) at room temperature.

## Author Contributions

All authors planned the experiment and discussed the data. The sample was fabricated by S.Y.; the measurement was performed by S.Y. W.Z. and H.D.; Z.S. and L.L. prepared the manuscript, and all authors reviewed it.

## Additional Information

**How to cite this article**: Yu, S. *et al*. Multilayer thin films with compositional PbZr_0.52_Ti_0.48_O_3_/Bi_1.5_Zn_1.0_Nb_1.5_O_7_ layers for tunable applications. *Sci. Rep.* **5**, 10173; doi: 10.1038/srep10173 (2015).

## Figures and Tables

**Table 1:** A comparison of the tunable characteristics of multilayer thin films with four PPBLs, PZT thin films and BZN thin films.

	***n***_***r***_	*ε**(0)***	***tanδ***	***E(kV/cm)***	***β(J/C***^***4***^***m***^*5*^)
**Four PPBLs**	53.3%	538	0.014	500	4.35×10^9^
**PZT**	57.8%	959	0.043	500	3.32×10^9^
**BZN**	6.5%	193	0.001	500	2.11×10^9^
